# Creation of a pandemic memory by tracing COVID-19 infections and immunity in Luxembourg (CON-VINCE)

**DOI:** 10.1186/s12879-024-09055-z

**Published:** 2024-02-09

**Authors:** Olena Tsurkalenko, Dmitry Bulaev, Marc Paul O’Sullivan, Chantal Snoeck, Soumyabrata Ghosh, Alexey Kolodkin, Basile Rommes, Piotr Gawron, Armin Rauschenberger, Carlos Vega Moreno, Clarissa P. C. Gomes, Anne Kaysen, Jochen Ohnmacht, Valerie E. Schröder, Lukas Pavelka, Guilherme Ramos Meyers, Laure Pauly, Claire Pauly, Anne-Marie Hanff, Max Meyrath, Anja Leist, Estelle Sandt, Gloria A. Aguayo, Magali Perquin, Manon Gantenbein, Tamir Abdelrahman, Jochen Klucken, Venkata Satagopam, Christiane Hilger, Jonathan Turner, Michel Vaillant, Joëlle V. Fritz, Markus Ollert, Rejko Krüger, on behalf of the CON-VINCE consortium and the ORCHESTRA working group, Marc Paul O’Sullivan, Chantal Snoeck, Soumyabrata Ghosh, Alexey Kolodkin, Basile Rommes, Piotr Gawron, Armin Rauschenberger, Carlos Vega Moreno, Clarissa P. C. Gomes, Anne Kaysen, Jochen Ohnmacht, Valerie E. Schröder, Lukas Pavelka, Guilherme Ramos Meyers, Laure Pauly, Anne-Marie Hanff, Max Meyrath, Anja Leist, Estelle Sandt, Gloria A. Aguayo, Magali Perquin, Tamir Abdelrahman, Jochen Klucken, Venkata Satagopam, Christiane Hilger, Jonathan Turner, Michel Vaillant, Joëlle V. Fritz, Geeta Acharya, Pinar Alper, Wim Ammerlaan, François Ancien, Ariane Assele-Kama, Christelle Bahlawane, Katy Beaumont, Nadia Beaupain, Lucrèce Beckers, Camille Bellora, Fay Betsou, Luc Biver, Sandie Boly, Dirk Brenner, Henry-Michel Cauchie, Eleftheria Charalambous, Emilie Charpentier, Estelle Coibion, Sylvie Coito, Delphine Collart, Manuel Counson, Brian De Witt, Antonelle Di Pasquale, Olivia Domingues, Claire Dording, Jean-Luc Dourson, Bianca Dragomir, Tessy Fautsch, Jean-Yves Ferrand, Thibault Ferrandon, Ana Festas Lopes, Guillaume Fournier, Laura Georges, Stéphane Gidenne, Enrico Glaab, Borja Gomez Ramos, Vyron Gorgogietas, Jérôme Graas, Valentin Groues, Wei Gu, Gael Hamot, Maxime Hansen, Linda Hansen, Lisa Hefele, Laurent Heirendt, Ahmed Hemedan, Estelle Henry, Margaux Henry, Eve Herkenne, Sascha Herzinger, Laetitia Huiart, Alexander Hundt, Judith Hübschen, Gilles Iserentant, Philipp Jägi, Piyapong Khurmin, Fédéric Klein, Tommy Klein, Stéphanie Kler, Pauline Lambert, Jacek Jaroslaw Lebioda, Sabine Lehmann, Marie Leick, Morgane Lemaire, Andrew Lumley, Annika Lutz, João Manuel Loureiro, Monica Marchese, Tainà Marques, François Massart, Patrick May, Maura Minelli, Alessandra Mousel, Maeva Munsch, Sophie Mériaux, Friedrich Mühlschlegel, Mareike Neumann, Trang Nguyen, Beatrice Nicolai, Leslie Ogorzaly, Christiane Olesky, Christian Penny, Achilleas Pexaras, Palma di Pinto, Marie France Pirard, Jean-Marc Plesseria, Armin Rauschenberger, Lucie Remark, Antonio Rodriguez, Kirsten Rump, Bruno Santos, Aurélie Sausy, Margaux Schmitt, Christiane Schmitt, Reinhard Schneider, Serge Schumacher, Alexandra Schweicher, Sneeha Seal, Jean-Yves Servais, Florian Simon, Amna Skrozic, Kate Sokolowska, Lara Stute, Hermann Thien, Stéphane Toll, Noua Toukourou, Christophe Trefois, Johanna Trouet, Nguyen Trung, Daniela Valoura Esteves, Charlène Verschueren, Maharshi Vyas, Claus Vögele, Cécile Walczak, Xinhui Wang, Femke Wauters, Bernard Weber, Emilie Weibel, Tania Zamboni

**Affiliations:** 1 Luxembourg Institute of Health, Strassen, Luxembourg; 2 University of Luxembourg, Esch-Belval, Luxembourg; 3 Centre Hospitalier de Luxembourg, Luxembourg, Luxembourg; 4 Maastricht University Medical Centre, Maastricht, The Netherlands; 5 Laboratoire National de Santé, Dudelange, Luxembourg

**Keywords:** Coronavirus (COVID-19), SARS-CoV-2, Prospective cohort study, Prevalence

## Abstract

**Background:**

During the COVID-19 pandemic swift implementation of research cohorts was key. While many studies focused exclusively on infected individuals, population based cohorts are essential for the follow-up of SARS-CoV-2 impact on public health. Here we present the CON-VINCE cohort, estimate the point and period prevalence of the SARS-CoV-2 infection, reflect on the spread within the Luxembourgish population, examine immune responses to SARS-CoV-2 infection and vaccination, and ascertain the impact of the pandemic on population psychological wellbeing at a nationwide level.

**Methods:**

A representative sample of the adult Luxembourgish population was enrolled. The cohort was followed-up for twelve months. SARS-CoV-2 RT-qPCR and serology were conducted at each sampling visit. The surveys included detailed epidemiological, clinical, socio-economic, and psychological data.

**Results:**

One thousand eight hundred sixty-five individuals were followed over seven visits (April 2020—June 2021) with the final weighted period prevalence of SARS-CoV-2 infection of 15%. The participants had similar risks of being infected regardless of their gender, age, employment status and education level. Vaccination increased the chances of IgG-S positivity in infected individuals. Depression, anxiety, loneliness and stress levels increased at a point of study when there were strict containment measures, returning to baseline afterwards.

**Conclusion:**

The data collected in CON-VINCE study allowed obtaining insights into the infection spread in Luxembourg, immunity build-up and the impact of the pandemic on psychological wellbeing of the population. Moreover, the study holds great translational potential, as samples stored at the biobank, together with self-reported questionnaire information, can be exploited in further research.

**Trial registration:**

Trial registration number: NCT04379297, 10 April 2020.

**Supplementary Information:**

The online version contains supplementary material available at 10.1186/s12879-024-09055-z.

## Introduction

The first case of the disease caused by the severe acute respiratory syndrome coronavirus 2 (SARS-CoV-2) was recorded on the 8th of December 2019 in Wuhan, China. The first case in Luxembourg was reported on 29th of February 2020 [1]. followed by a rapid spread of the virus across the country.

Cohort studies allow accumulating epidemiological evidence from representative samples of population and to advance medical knowledge related to SARS-CoV-2 infection. Although several nationwide population-based studies of COVID-19 are available globally [2–7]. detailed knowledge about the dynamics and spread of the infection and immune status in the general population are still limited. A longitudinal systematic observation of a representative sample of the population irrespective of clinical symptoms may help to clarify different aspects of the novel coronavirus infection.

Luxembourg is a landlocked country in the middle of Europe, with 634,730 inhabitants as of 1st January 2021 [8]. bordering Belgium, France, and Germany. It is closely connected to its neighbours, and more than 200,000 cross border workers commute daily to Luxembourg. Here we consider Luxembourg as a model for providing comprehensive information about the different aspects of SARS-CoV-2 infection and COVID-19 pandemic on a representative national level that was utilised to address the following Research questions:

(i) Are there significant differences in SARS-CoV-2 infection prevalence that can be observed by the end of the study among different socio-demographic subgroups?

(ii) Is there a clear difference in terms of IgG anti-S positivity between vaccinated and non-vaccinated individuals after adjustment for the SARS-CoV-2 infection status?
(iii) Did restrictive measures have an effect on the mental health of the Luxembourgish population, namely, on depression, anxiety, stress and loneliness levels?

In this manuscript, we present a descriptive summaries of the CON-VINCE study, estimate the point and period prevalence of the SARS-CoV-2 infection in the Luxembourgish population, examine specific immune response to SARS-CoV-2 infection and vaccination, and the impact of the containment measures on psychological wellbeing at a population level.

## Materials and Methods

### Study design

The CON-VINCE study (COvid-19 National survey for assessing VIral spread by Non-affected CarriErs) is a longitudinal observational study collecting self-reported survey information via online tools and biosamples via local private laboratories. It started in April 2020 and followed participants over seven visits until June 2021. A list of collected data and biosamples is provided in Table 1.

**Table 1:** Data and sample collection across visits

	Baseline	Follow-up					
*Visit*	0	1	2	3	4	5	6
*Week*	0	2	4	6	8	48	52
*Month*	Apr 2020	May 2020	May 2020	Jun 2020	Jun 2020	Mar 2021	Apr – Jun 2021
Questionnaire							
Inclusion / Exclusion criteria	•						
SARS-CoV-2 exposure and infection	•	•	•	•	•	•	•
Epidemiological factors	•	•	•	•	•	•	•
Demographics	•					•	•
Education / Professional background	•	•	•	•	•	•	•
Home and social contacts	•	•	•	•	•	•	•
Socio-economic status ^a^	•	•	•	•	•	•	•
Comorbidities	•		•	•	•	•	•
Current medications	•					•	•
Respiratory symptoms onset	•	•	•	•	•		•
Signs and symptoms	•	•	•	•	•	•	•
Symptoms onset and initial clinical signs of SARS-CoV-2 infection	•						
SARS-CoV-2 vaccine						•	•
Environmental conditions at home with a diagnosis of COVID-19	•	•	•	•	•		•
Contact tracing information	•	•	•	•	•		•
Behavioural analysis during COVID-19 pandemic ^b^	•	•	•	•	•	•	•
*Psychological questionnaires*							
CES-D^a^	•	•	•	•	•	•	•
GAD-7^a^	•	•	•	•	•	•	•
UCLA short version^a^	•	•	•	•	•	•	•
PSS-4^a^	•	•	•	•	•	•	•
BRS^a^	•	•	•	•	•	•	•
BFI-10	•	•	•	•	•		•
Major life events							
Childhood Trauma Questionnaire					•		
Samples	•	•	•	•	•		•
Serum	•	•	•	•	•		•
Plasma	•	•	•	•	•		•
Buffy Coat	•	•	•	•	•		•
PBMCs							•
Combined naso/oropharyngeal swab	•	•	•	•	•		•
Stool sample^a^	•	•	•	•	•		•
SARS-CoV-2 diagnostics	•	•	•	•	•		•
IgA, IgG-S anti-SARS-CoV-2 antibodies measurements	•	•	•	•	•		•
IgG-N anti-SARS-CoV-2 antibodies measurements							•
SARS-CoV-2 neutralisation capacity of antibodies							•
RT-qPCR against SARS-CoV-2	•	•	•	•	•		•

*CES-D* Center for Epidemiologic Studies - 20-item Depression scale, *GAD-7 *Generalised Anxiety Disorder - 7-item scale, *UCLA* short version - University of California Los Angeles 3-item Loneliness scale, *PSS-4 *Perceived Stress 4-item Scale, *BRS *Brief Resilience Scale, *BFI-10 *Big Five personality traits 10-item scale, *PBMCs *Peripheral blood mononuclear cell

^a^Optional collection of data (extended survey) / samples

^b^Also included compliance to government recommendations during pandemic

### Visits

Capturing the dynamics and the impact of virus spread over time, participants were followed-up over twelve months. CON-VINCE participants completed a survey and donated biological material bi-weekly, from April 2020 to June 2020 (Visits 0–4) and at the annual follow-up visit 6. One-three months before the annual visit, only online survey information was collected (Visit 5).

Participants were tested for SARS-CoV-2 by RT-qPCR and anti-SARS-CoV-2 serology at all sampling visits. Data and samples collected across the visits are presented in Table 1. Additional sample collections one year after the start of the pandemic and vaccine implementation, allowed to investigate breakthrough infections and vaccine escape [9].

A subset of the CON-VINCE cohort was followed-up within the framework of the European ORCHESTRA consortium in which 15 countries collaborate for deeper investigation of different aspects of the COVID-19 pandemic prospectively and retrospectively, including prevention and treatment of SARS-COV-2 infections [10].

### Cohort description

A representative sample of the adult Luxembourgish population was included in the study. All individuals except for severely affected COVID-19 patients requiring hospitalisation were included, irrespective of their SARS-CoV-2 infection status, and followed-up longitudinally even if they had been hospitalised for COVID-19 at some point after the baseline. By design, the CON-VINCE cohort included non-infected participants, allowing for comparison of individual pre- and post-infection clinical characteristics and serological status.

Initially, participants received via email detailed electronic Subject Information Sheet and an electronic Informed Consent Form, and were asked to provide personal identifiable information through a secure online interface. Then, participants meeting inclusion criteria, received a link to complete online questionnaires. Captured data was linked to a study pseudonym. Telephone support for the questionnaire was offered. Participants were invited for SARS-CoV-2 diagnostics to approved local laboratories within a week after the online survey completion.

### Study Questionnaires

Participants could complete the full online survey (extended survey) or, alternatively, a mandatory version (minimal survey). The minimal survey included basic epidemiological and clinical data required for the prevalence study on SARS-CoV-2 infections and immunity. The extended survey was based on the International Severe Acute Respiratory and emerging Infection Consortium (ISARIC) [11]. covering wide range of domains, including socio-economic factors and psychological wellbeing (Table 1). Minimizing potential language bias, all questionnaires were provided in four languages (English, French, Portuguese, and German). Supplementary Table 1 provides detailed information captured in the online survey.

The psychological test battery included: Center for Epidemiologic Studies Depression Scale (CES-D Scale), a 20-item measure assessing depressive symptoms (total range: 0—60) [12, 13]. Generalised Anxiety Disorder 7-item scale (GAD-7)—screening tool for anxiety (total range: 0—21) [14, 15]. University of California Los Angeles (UCLA) Loneliness Scale short version, a 3-item measure assessing the extend a person feels disconnected from others (total range: 3—9) [16, 17]. Perceived Stress Scale 4 (PSS-4), 4 item version of the classic stress assessment instrument (total range: 0—16) [18, 19]. Brief Resilience Scale (BRS) used to assess the perceived resilience scored on a Likert-Scale (total range: 1—5) [20]; Big Five Inventory 10 (BFI-10), a 10-item scale measuring the personality traits: Extraversion, Agreeableness, Conscientiousness, Neuroticism, and Openness (range: 1—5 for each) [21].

Health status evaluation focused on 29 symptoms associated with SARS-CoV-2 (summarised in Supplementary Table 2) and comorbidities.

### Sample collection and processing

Collection of blood samples and swabs was conducted by private partner laboratories across the country (Laboratoires Réunis, Ketterthill, BioneXt), Laboratoire National de la Santé (LNS), and the Luxembourg Institute of Health (LIH) Clinical and Epidemiological Investigation Center (CIEC) team, some providing home collection services (Picken Doheem). Daily transports from collection sites to the Integrated Biobank of Luxembourg (IBBL) avoided processing delays and guaranteed biosample integrity. Swab samples RT-qPCR were performed at the LNS or the Department of Infection and Immunity (DII) of LIH. Samples were sequenced to determine the virus variant if sufficient sample was present. Serology was performed at the DII.

### RT-qPCR

At the LNS, SARS-CoV-2 detection was carried out using the Allplex 2019 n-CoV Assay (Seegene) targeting RdRP, N and E gene. Samples in which only the SARS-CoV-2 RdRP or the N gene was detected were retested in duplicate at LIH by RT-qPCR in house assays targeting the E gene and N gene (N1 target; CDC) and the FTD SARS-CoV-2 commercial assay (Fast Track Diagnostics, Esch-sur-Alzette, Luxembourg; N and ORF1ab genes). All N gene RT-qPCR positive samples, were considered positive when Cq < 40 was obtained in at least one replicate for 2 viral genes in 2 independent confirmatory RT-qPCR [16, 17].

### Serology

Anti-Spike (S) SARS-CoV-2 IgA and IgG and anti-Nucleocapsid (N) IgG were determined by enzyme-linked immunosorbent assay (ELISA) kits (Euroimmun), according to the manufacturer’s instructions using provided positivity thresholds. Samples with intermediate OD ratios (≥ 0.8, < 1.1) were judged borderline positive for subsequent analyses.

#### Definition of prevalence and infection

Within CON-VINCE, two types of prevalence are estimated. The proportion of individuals with an active infection identified via an RT-qPCR, conducted in the framework of the study at each particular visit (point prevalence) and the proportion of individuals infected in the period between the first positive case in Luxembourg and the date of the considered visit (period prevalence).

Participants were identified as infected in a case of a positive RT-qPCR or/and presence of IgG-S to SARS-CoV-2 prior to vaccination or/and presence of IgG-N to SARS-CoV-2 or/and self-reported positive SARS-CoV-2 test result (RT-qPCR in > 95% cases; other tests < 5% cases) observed at least at one of the visits.

### Data management

**Fig. 1:**
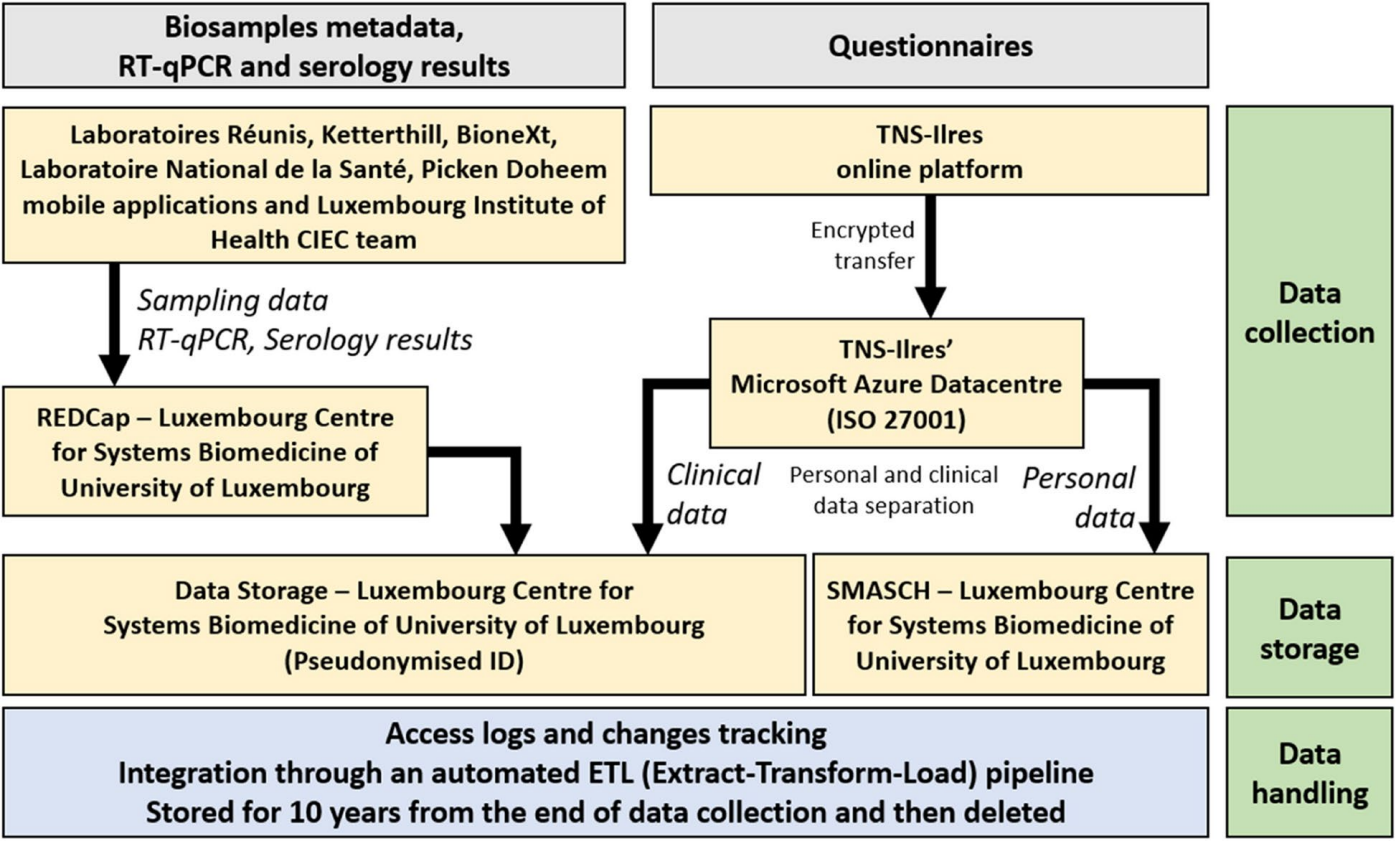
Data management

Personal identification information was stored in SMASCH, a separate secured platform [18]. A dedicated REDCap instance was deployed to store the pseudonymised biosample metadata, RT-qPCR and serology results (Fig. 1). Positive RT-qPCR participants were contacted and, informed about their status, as well as provided with the instructions on further actions.

### Statistical analysis

a Sample Size Calculation

Given the rapidly changing incidence of those infected as of 10th April 2020, when the study protocol was established, a 50% COVID-19 true prevalence was assumed, resulting in a required minimum sample size of 1,537 participants, assuming 2.5% accuracy.

The sampling frame consisted of adult inhabitants of Luxembourg, chosen from a web panel of 18,000 individuals, provided by the survey company (TNS-Ilres). The selected sampling strategy for the sample for the representativeness of the general Luxembourgish population was stratified by gender, age categories (10-year bins from the age of 18 and over) and constituencies. Within strata, a deterministic random bit generator was used to apply an equal allocation probability, proportional to the strata size, without replacement.

To compensate for non-response and potential dropouts during the study, 1,865 individuals were allowed to participate.

b Descriptive analysis

Categorical variables were summarised as counts (n) and proportions (%). Normally distributed continuous variables were described by means and standard deviations (SD). Non-normally distributed continuous variables were summarised by medians and interquartile ranges (IQR).

iii. Inferential analysis

We assumed Missing At Random (MAR) missing data pattern for all our analysis.

Point prevalence and sero-prevalence of SARS-CoV-2 infection, as well as the cumulative number of infected participants (period prevalence) were estimated at each visit (except for V5) via applying post-stratification. Weighing was used to ensure representativeness of summaries, and adjusting for the non-attendants.

In order to estimate prevalence of COVID-19 symptoms at visit 6, weighting through post-stratification was implemented, resulting in the identical strata proportions in the weighted sample and the actual population (age group by gender).

A subgroup analysis was performed for SARS-CoV-2 infection period prevalence. The age of infected individuals was compared to the age of non-infected individuals via a Mann–Whitney-U Test. Gender was compared between the two groups through a Chi-Squared test. Employment status and education level were compared using Fisher’s exact tests. Bonferroni correction was applied to the results of the tests, to account for multiplicity.

A comparison of the sero-prevalence between the vaccinated and non-vaccinated individuals with a history of SARS-CoV-2 infection has been performed through a Chi-Squared test.

Four of the psychological scale scores were dichotomised in order to obtain clinically meaningful interpretation of those. Four binary variables were obtained with the cut-offs meaning Depression (CES-D >  = 16), Mild anxiety (GAD >  = 5), High loneliness (UCLA >  = 6), and High stress level (PSS-4 >  = 6). The effect of the containment measures on mental health was evaluated through a series of comparisons of these four variables at the baseline visit against visits 5 and 6. Only the individuals with data available at both visits were included in comparisons. McNemar’s tests were used to perform comparisons, followed by Bonferroni adjustment.

To investigate potential reasons for respondent attrition in the study, the age, gender and education level of respondents who attended the last study visit, were compared to those who did not participate in this visit. Age (continuous) was compared between dropouts and non-dropouts via a Mann–Whitney-U Test. Gender and Education level variables were compared by Chi-Squared tests. Bonferroni correction was applied to the results of the tests.

## Results

### Population characteristics

**Fig. 2:**
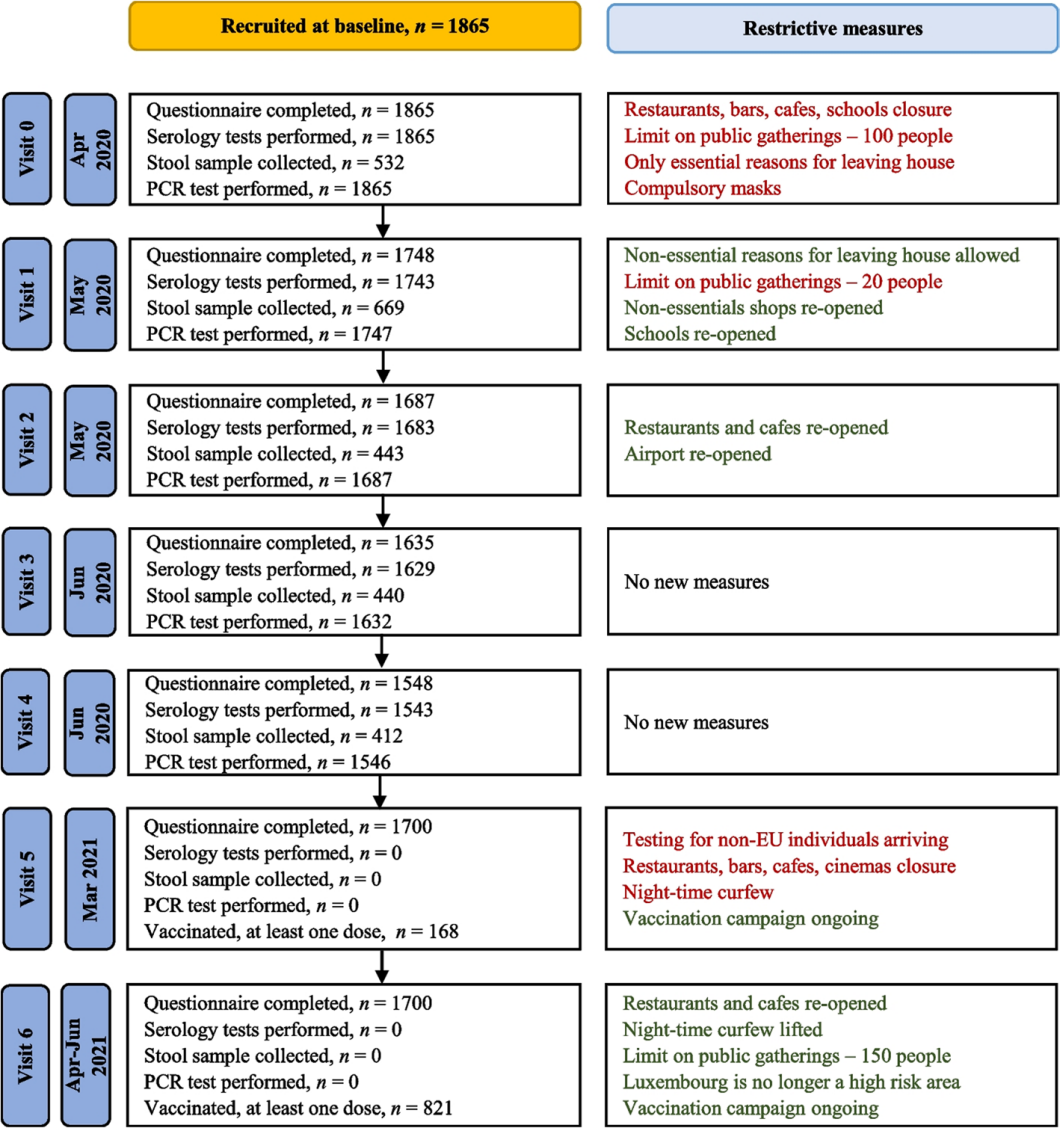
Flowchart describing each visit of the study

Between April 15th and May 4th 2020, *n* = 1,865 Luxembourg residents joined the CON-VINCE study. At the baseline visit, 1,865 participants completed the questionnaire, were tested via RT-qPCR for SARS-CoV-2 infection and provided blood samples. A flowchart describing the number of respondents at each stage of the study is presented in Fig. 2.

Core demographic characteristics of the cohort are summarised in Table 2. Most of the participants were in the age range of 30–69 years (76%), with 14% younger, and 10% older. The mean age at baseline was 48 years, while the two dominant genders were represented in similar proportions (51% females and 49% males). As for other socio-demographic characteristics, 55% of participants were married, 60% employed or self-employed, 23%—retired. Detailed demographic characteristics of the cohort are provided in Table 2.

**Table 2:** Core demographic characteristics

	Visit 0	Visit 1	Visit 2	Visit 3	Visit 4	Visit 5	Visit 6
*Month*	Apr 2020	May 2020	May 2020	Jun 2020	Jun 2020	Mar 2021	Apr-Jun 2021
*N*	1865	1748	1687	1635	1548	1700	1578
Age, *Mean (SD)*	48 (15)	48 (15)	49 (15)	49 (15)	49 (15)		49 (15)
**Age Group, ** ***n (%)***							
18-29	259 (14)	222 (13)	211 (13)	196 (12)	183 (12)		180 (11)
30-39	368 (20)	339 (19)	324 (19)	302 (18)	284 (18)		278 (18)
40-49	400 (21)	379 (22)	362 (21)	347 (21)	339 (22)		351 (22)
50-59	374 (20)	360 (21)	343 (20)	344 (21)	322 (21)		311 (20)
60-69	284 (15)	274 (16)	272 (16)	274 (17)	257 (17)		285 (18)
70-79	164 (9)	161 (9)	159 (9)	158 (10)	150 (10)		137 (9)
80-85	16 (1)	13 (1)	16 (1)	14 (1)	13 (1)		12 (1)
Unknown							24 (2)
**Gender, ** ***n (%)***							
Female	946 (50.7)	885 (50.6)	852 (50.5)	826 (50.5)	780 (50.4)		781 (49.5)
Male	917 (49.2)	861 (49.3)	833 (49.4)	807 (49.4)	766 (49.5)		795 (50.4)
Diverse/ Unknown	2 (0.1)	2 (0.1)	2 (0.1)	2 (0.1)	2 (0.1)		2 (0.1)
**Education Level, ** ***n (%)***							
University degree	765 (41)	716 (41)	692 (41)	660 (40)	632 (41)		730 (46)
Secondary Education - Technical system	463 (25)	434 (25)	415 (25)	406 (25)	381 (25)		405 (26)
Secondary Education - Classical system	295 (16)	272 (16)	263 (16)	257 (16)	239 (15)		275 (17)
Other type of degree	246 (13)	238 (14)	234 (14)	228 (14)	217 (14)		117 (7)
No formal degree^a^	54 (3)	51 (3)	49 (3)	49 (3)	47 (3)		22 (1)
Fundamental Education	42 (2)	37 (2)	34 (2)	35 (2)	32 (2)		29 (2)
**Electoral District, ** ***n (%)***							
Centre	659 (35)	616 (35)	583 (35)	576 (35)	540 (35)		
South	672 (36)	631 (36)	617 (37)	588 (36)	563 (36)		
North	290 (16)	271 (16)	260 (15)	251 (15)	235 (15)		
East	244 (13)	230 (13)	227 (13)	220 (13)	210 (14)		
**Employment status, ** ***n (%)***							
Full-time employed	824 (44)	753 (43)	721 (43)	706 (43)	673 (43)	772 (45)	706 (45)
Part-time employed	233 (12)	235 (13)	234 (14)	223 (14)	213 (14)	214 (13)	197 (12)
Self-employed or working for own family business	66 (4)	57 (3)	56 (3)	57 (3)	52 (3)	59 (3)	60 (4)
In vocational training/ retraining/ education	73 (4)	67 (4)	63 (4)	53 (3)	49 (3)	60 (4)	42 (3)
In retirement or early retirement	438 (23)	425 (24)	421 (25)	423 (26)	396 (26)	427 (25)	416 (26)
Looking after home or family	91 (5)	82 (5)	77 (5)	73 (4)	72 (5)	82 (5)	70 (4)
Parental leave	23 (1)	22 (1)	21 (1)	13 (1)	15 (1)	13 (1)	12 (1)
Permanently sick or disabled	13 (1)	8 (0)	10 (1)	10 (1)	12 (1)	13 (1)	15 (1)
Unemployed	26 (1)	29 (2)	26 (2)	25 (2)	21 (1)	14 (1)	13 (1)
Other employment status	78 (4)	70 (4)	58 (3)	53 (3)	46 (3)	47 (3)	47 (3)
**Marital status, ** ***n (%)***							
Single	404 (22)	364 (21)	346 (21)	322 (20)	305 (20)	353 (21)	325 (21)
Married	1020 (55)	972 (56)	947 (56)	934 (57)	895 (58)	955 (56)	895 (57)
Registered partnership	198 (11)	182 (10)	174 (10)	164 (10)	150 (10)	186 (11)	156 (10)
Divorced	150 (8)	144 (8)	131 (8)	131 (8)	121 (8)	116 (7)	129 (8)
Widowed	46 (2)	43 (2)	46 (3)	45 (3)	42 (3)	46 (3)	47 (3)
Other marital status	47 (3)	43 (2)	43 (3)	39 (2)	35 (2)	44 (3)	26 (2)

^a^The participants declaring “no formal degree” are adults (21-80 years old) who do not have a guardian/authorised representative and are in full capacity to understand and complete the consent forms independently. More than that, 60% of such participants are above the age of 50 at baseline, indicating that unconventional ways of schooling might have been used

### SARS-CoV-2 infection prevalence

Point prevalence at the baseline visit was 0.6% (12 of 1,865 subjects with positive SARS-CoV-2 RT-qPCR). At the annual follow-up visit 7 of 1,329 (0.5%) had a positive RT-qPCR result.

Serological analysis on all 1,865 participants at the baseline visit identified 39 participants (2.1%) with positive/borderline IgG anti-S antibodies against SARS-CoV-2. At visit 6, IgG anti-N and IgG anti-S antibodies were measured for 1,324 samples. 8% (111/1324 participants presented with anti-N IgG titres, while 49% (644/1324) displayed anti-S IgG titres.

Participants also self-reported positive SARS-CoV-2 tests performed outside the study since June 2020. By visit 6, 10% of participants (188/1,865), had declared a positive test result. Detailed prevalence at each study visit are presented in Table 3.

**Table 3:** Point prevalence, sero-prevalence and sample frequencies

Visit	Visit 0	Visit 1	Visit 2	Visit 3	Visit 4	Visit 5	Visit 6
Month	Apr 2020	May 2020	May 2020	Jun 2020	Jun 2020	Mar 2021	Apr-Jun 2021
RT-qPCR tests performed, *n*	1,865	1,747	1,687	1,632	1,546	0	1,329
RT-qPCR positive, *n (%)* (Point prevalence)	12 (0.6%)	2 (0.1%)	1 (0.1%)	2 (0.1%)	0 (0.0%)		7 (0.5%)
RT-qPCR positive, weighted, *n (%)* *(Weighted point prevalence)*	3,773 (0.7%)	674 (0.1%)	320 (0.1%)	549 (0.1%)	0 (0%)		2,734 (0.5%)
IgG-S serology tests performed, *n*	1,865	1,743	1,683	1,629	1,543	0	1,324
IgG-S positive/borderline, *n (%)*	39 (2.1%)	39 (2.2%)	43 (2.6%)	37 (2.3%)	39 (2.5%)		644 (48.6%)
IgG-S positive/borderline, weighted, *n (%)*	11,199 (2.2%)	12,253 (2.4%)	13,586 (2.7%)	12,247 (2.4%)	13,664 (2.7%)		232,689 (45.3%)
Population corresponding to weighted sample ^a^, *N*	506,569	506,569	506,569	506,569	506,569		513,736
Official cumulative number of cases, *n (%)* ^b^	3,139 (0.6%)	3,197 (0.6%)	3,228 (0.6%)	3,301 (0.7%)	3,577 (0.7%)		70,406 (13.7%)
Cumulative IgG-S sero-prevalence, including vaccinated (as a proportion of the whole cohort), *n (%)*	11,199 (2.2%)	12,243 (2.4%)	13,213 (2.6%)	13,416 (2.6%)	13,636 (2.7%)		176,381 (34.3%)

^a^Figures representing the total population of Luxembourg of age 18 or older in the years 2020 and 2021 were taken from the website of the governmental statistics service of Luxembourg, STATEC [12]

^b^Cumulative number of SARS-CoV-2 positive cases registered in Luxembourgish residents, according to RT-qPCR test results, considered on the date when blood sampling was conducted for the last participant at each CON-VINCE visit. These values were obtained directly from the Luxembourgish Data Platform website of Government of Grand Duchy of Luxembourg [13]

We observed that by the end of the study, June 2021, the period prevalence of SARS-CoV-2 infection was 15% in the weighted sample, matching the officially reported number of cases, as a proportion of adult population – 14%. In contrast to other studies, here we observed identical prevalence thanks to the broad testing regimen in Luxembourg that captured the actual infection prevalence at a national level.

To assess if specific sociodemographic characteristics are associated with infection risk, we analysed data at visit 6. Comparing the demographic characteristics of participants, who were infected (definitions provided in methods) by the end of the study, compared to those who were not (Table 4), revealed that infected individuals were significantly younger, however, the difference was not significant after multiplicity adjustment. Neither gender, age, employment status, nor education level was associated with altered risk.

**Table 4:** Comparison of infected and non-infected individuals at visit 6

	**Non-infected**	**Infected**	**Unadjusted ***p*-value	**Adjusted** *p*-value
	**Mean (SD)**	**Mean (SD)**		
**Age, years**	49 (15)	47 (15)	0.040*	0.160
**Gender**	**Freq. (Proportion)**	**Freq. (Proportion)**	0.667	1.000
**Male**	677 (51%)	118 (49%)		
**Female**	659 (49%)	122 (51%)		
**Education level**	**Freq. (Proportion)**	**Freq. (Proportion)**	0.684	1.000
**University degree**	612 (46%)	118 (49%)		
**Secondary Education - Technical system**	346 (26%)	59 (25%)		
**Secondary Education - Classical system**	230 (17%)	45 (19%)		
**Other type of degree**	104 (8%)	13 (5%)		
**No formal degree**	20 (1%)	2 (1%)		
**Fundamental Education**	26 (2%)	3 (1%)		
**Employment status**	**Freq. (Proportion)**	**Freq. (Proportion)**	0.112	0.449
**Full-time employed**	593 (44%)	113 (47%)		
**In retirement or early retirement**	367 (27%)	49 (20%)		
**Part-time employed**	160 (12%)	37 (15%)		
**Looking after home or family**	57 (4%)	13 (5%)		
**Self-employed or working for own family business**	53 (4%)	7 (3%)		
**Other, please specify**	43 (3%)	4 (2%)		
**In vocational training/retraining/education**	31 (2%)	11 (5%)		
**Permanently sick or disabled**	13 (1%)	2 (1%)		
**Unemployed**	10 (1%)	3 (1%)		
**Parental leave**	11 (1%)	1 (0%)		

* Statistical significance at α=0.05 confidence level

By the end of the study, 821 respondents had declared that they had received at least one dose of an anti-SARS-CoV-2 vaccine, corresponding to 52% of those who attended the visit, and 44% of the total study population. These numbers are close the officially recorded vaccination rate of 50% (adults who received at least one dose).

In 709 of 821 vaccinated participants, samples at visit 6 were available, allowing assessment of IgG anti-S status. From those, 556 (78%) had positive and 153 (22%) had negative IgG-S titres. Considering infected individuals only, 85 out of 93 (91%) vaccinated participants had positive IgG-S antibodies, compared to 77 out of 113 (68%) in the non-vaccinated group. The difference in sero-positivity between those two groups was significant at 5% confidence level (*p*-value < 0.001).

### COVID-19 symptom prevalence

At visit 6, the prevalence of symptoms typically associated with COVID-19 was 49.2% after weighting. The five most prevalent symptoms in infected individuals were anxiety (34.4%), depression (23.8%), sleep difficulties (19.6%), fatigue (15.6%), and joint pain (11.5%). Never infected participants presented with similar prevalence of symptoms, except for fatigue (5.6%). Supplementary Table 2 provides more information on COVID-19 symptoms prevalence.

### Population mental health during the COVID-19 pandemic

In CON-VINCE, the psychological impact of the SARS-CoV-2 pandemic was evaluated by assessing a wide range of psychological scales (Table 5). The median values of CES-D, GAD-7, and UCLA Loneliness scales remained stable between visits 0 and 6, while the median score in the PSS-4 scale decreased between baseline and visit 6 by one unit.

**Table 5:** Psychological assessment questionnaires

**Visit**	**Visit 0**		**Visit 1**		**Visit 2**		**Visit 3**		**Visit 4**		**Visit 5**		**Visit 6**	
**Month**	**Apr 2020**		**May 2020**		**May 2020**		**Jun 2020**		**Jun 2020**		**Mar 2021**		**Apr-Jun 2021**	
**Psychological scale**	***n***	**Median [IQR]**	***n***	**Median [IQR]**	***n***	**Median [IQR]**	***n***	**Median [IQR]**	***n***	**Median [IQR]**	***n***	**Median [IQR]**	***n***	**Median [IQR]**
**CES-D**	1865	8 [4; 14]	1748	7 [4; 13]	1687	6 [3; 12]	1635	5 [2; 11]	1548	6 [2; 11]	1694	9 [5; 17]	1570	8 [3; 14]
**GAD** ***-*** **7**	1847	2 [1;5]	1718	2 [0; 4]	1645	2 [0; 4]	1581	1 [0; 4]	1492	1 [0; 4]	1696	3 [0; 6]	1572	2 [0; 5]
**UCLA short version**	1865	4 [0; 5]	1748	4 [3; 5]	1687	4 [3; 5]	1635	4 [3; 5]	1548	4 [3; 5]	1696	5 [4; 6]	1573	4 [3; 5]
**PSS-4**	1865	5 [0; 7]	1748	4 [2; 6]	1687	4 [2; 6]	1635	3 [2; 6]	1548	3 [1; 6]	1694	5 [2; 7]	1569	4 [2; 7]
**BRS**	1848	3.8 [3.3; 4.3]	1732	3.8 [3.3; 4.3]	1673	3.8 [3.3; 4.3]	1622	3.8 [3.3; 4.3]	1536	3.8 [3.3; 4.3]	1693	3.7 [3.2; 4.2]	1568	3.8 [3.2; 4.2]
**BFI Extraversion**	1853	3.5 [3; 4]	1736	3.5 [3; 4]	1676	3.5 [3; 4]	1625	3.5 [3; 4]	1539	3.5 [3; 4]	0		1578	3.5 [3; 4]
**BFI Conscientiousness**	1853	4 [3.5; 4.5]	1736	4 [3.5; 4.5]	1676	4 [3.5; 4.5]	1625	4 [3.5; 4.5]	1539	4 [3.5; 4.5]	0		1578	4 [3.5; 4.5]
**BFI Agreeableness**	1853	3.5 [3; 4]	1736	3.5 [3; 4]	1676	3.5 [3; 4]	1625	3.5 [3; 4]	1540	3.5 [3; 4]	0		1578	3.5 [3; 4]
**BFI Neuroticism**	1854	2.5 [2; 3.5]	1737	2.5 [2; 3.5]	1677	2.5 [2; 3]	1626	2.5 [2; 3]	1540	2.5 [2; 3]	0		1578	2.5 [2; 3.5]
**BFI Openness**	1846	3.5 [3; 4]	1730	3.5 [3; 4]	1669	3.5 [3; 4]	1619	3.5 [3; 4]	1533	3.5 [3; 4]	0		1578	3.5 [3; 4]

*CES-D* Center for Epidemiologic Studies - 20-item Depression scale, *GAD-7* Generalized Anxiety Disorder - 7-item scale, *UCLA* short version - University of California Los Angeles 3-item Loneliness scale, *PSS-4* Perceived Stress 4-item Scale, *BRS* Brief Resilience Scale, *BFI-10* Big Five personality traits 10-item scale

To assess the impact of strict public health measures we compared psychological scale data from the last questionnaire collected under strict public health measures (visit 5) and when most of the restrictive public health measures were lifted (visit 6).

Depression, anxiety, loneliness and stress were increased significantly (adjusted *p*-values < 0.05) by visit 5. However, by visit differences to the baseline were no longer detectable. Table 6 provides detailed information on psychological scale scores at different time points according to defined cut-offs.

**Table 6:** Comparison of psychological scale and corresponding characteristics between baseline visit (visit 0) and the follow-ups (visits 5 & 6)

**Psychological scale and corresponding characteristic**	**Visit 0**	**Visit 5**	***p*** **-value**	**Adjusted ** ***p*** **-value**	**Visit 0**	**Visit 6**	***p*** **-value**	**Adjusted ** ***p*** **-value**
CES-D - number of individuals, *N*	1694		<0.001**	<0.001**	1570		0.058	0.466
Depressed, *n (%)*	342 (21%)	486 (29%)			309 (20%)	341 (22%)		
Not depressed, *n (%)*	1352 (79%)	1208 (71%)			1261 (80%)	1229 (78%)		
*GAD-7*- number of individuals, *N*	1680		0.003**	0.028*	1557		0.294	1.000
Anxious, *n (%)*	100 (6%)	139 (8%)			92 (6%)	105 (7%)		
Not anxious, *n (%)*	1580 (94%)	1541 (92%)			1465 (94%)	1452 (93%)		
UCLA short version - number of individuals, *N*	1696		<0.001**	<0.001**	1573		0.007**	0.059
Lonely, *n (%)*	345 (20%)	510 (30%)			322 (20%)	370 (24%)		
Not lonely, *n (%)*	1351 (80%)	1186 (70%)			1251 (80%)	1203 (76%)		
PSS-4 - number of individuals, *N*	1694		<0.001**	<0.001**	1569		0.601	1.000
Stressed, *n (%)*	623 (37%)	725 (43%)			566 (36%)	554 (35%)		
Not stressed, *n (%)*	1071 (63%)	969 (57%)			1003 (64%)	1015 (65%)		

The following cut-offs have been used in order to dichotomise the numeric psychological scales’ scores into a binary clinical outcome: *CES-D<16 *Not depressed, *CES-D>=16 *Depressed, *GAD<5 *Not anxious, *GAD>=5 *Anxious, *UCLA* *short version<6* Not lonely, *UCLA short version>=6 *Lonely, *PSS-4<6 *Not stressed, *PSS-4>=6 *Stressed

* Statistical significance at *α*=0.05 confidence level

** Statistical significance at *α*=0.01 confidence level

### Study adherence

Adherence remained high throughout the study. The number of participants completing the questionnaire varied between visits with a decrease of 17% from visit 0 (*n* = 1,865) to visit 4 (*n* = 1,548), an increase at visit 5 (attrition 9%; *n* = 1,700), with attrition rate of 15% at the final visit 6 (*n* = 1,578).

Fifteen participants (0.8%) withdrew from the study. Reasons for withdrawal from the study were not formally documented. Comparing between dropouts and non-dropouts at visit 6 demonstrated a statistically significant age difference (*p*-value < 0.001) between the two groups (Supplementary Table 3), with dropouts significantly younger (mean age = 43) than those who completed the last visit (mean age = 49).

## Discussion

Within a short time frame, we established a nationwide representative cohort to follow SARS-CoV-2 infection in the Luxembourgish population with comprehensive clinical, epidemiological, serological and psychosocial data collection. The longitudinal design of the CON-VINCE study allowed to track the build-up of immunity against SARS-CoV-2 over time and thereby shaped a pandemic memory. The study design allowed for a careful monitoring of seroconversion and antibody levels over time. In depth analysis of the collected biosamples will allow us a better interpretation of evolving antibody responses.

Overall, 23 RT-qPCR positive participants were identified during the study. The largest number was captured at visit 0, with lower frequencies observed at visits 1–5, followed by a jump at visit 6. This dynamic can be explained by the strict public health measures, maintained during the visits 1–5.

Weighted seroprevalence of IgG anti-s antibodies increased from 2.2% in April–May 2020 to 45.3% in April-June 2021, which is mainly due to the success of the vaccination campaign in Luxembourg (Fig. 2).

In the CON-VINCE cohort, the risk of infection was similar between all participants regardless of gender, age, employment status and education level. This may be explained by consistent adherence to recommended preventive and restrictive measures across different demographic groups.

From 188 individuals declaring a positive test result detecting SARS-CoV-2 infection conducted outside of the study since June 2020, 21 (11%) could not be confirmed as exposed by serology. This could be due to recall bias for some of the participants, leading in over-reporting of true exposure [22–24]. Alternatively, it may indicate that either antibody response was insufficient [25, 26]. or that fast antibody waning occurred. More frequent testing in future studies could help to capture the moment of exposure of all infected participants [27]. Alternatively, test based assessment of virus exposure should be prioritized during study testing, rather than self-reported data.

At the last visit of the study, among vaccinated participants of the cohort, 78% had positive or borderline positive IgG anti-S titres. Notably, among those who were infected, the vaccinated group demonstrated a significantly higher sero-prevalence of 91%, compared to 68% in the non-vaccinated group, emphasising the efficacy of vaccination in promoting a robust IgG-S antibody response even in infected individuals.

Between fully vaccinated participants who had IgG-S antibodies levels recorded at visit 6, a more apparent immunological response was observed to mRNA vaccines (Pfizer-BioNTech or Moderna) – 176/180, corresponding to 98%, compared to Vector vaccines (1 dose of Johnson&Johnson or 2 doses AstraZeneca) – 28/41, corresponding to 68%. However, when considering two doses as fully vaccinated for the Johnson&Johnson vaccine, 21/22 (95%) individuals vaccinated with vector machines demonstrated sero-positivity, reaching similar sero-positivity as for mRNA vaccines. Our findings confirm the effectiveness of both types of vaccines in terms of increasing IgG-S antibodies titres.

Interestingly, similar symptoms were reported by non-infected compared to infected participants, except for fatigue, which was less prevalent (5.6% versus 7.2%). This can be explained by the psychological impact of the pandemic, such as increased awareness about COVID-19 symptoms and by other health conditions, not related to SARS-CoV-2 infection [28, 29]. These findings highlight the phenomenon of presence of COVID-19-related symptoms among individuals, irrespective of their infection status, emphasising the importance of addressing and managing these symptoms in both groups.

In the CON-VINCE cohort, we observed a significant increase in depression, anxiety, loneliness and stress at the visit 5, when the containment measures were the strictest. While the negative effects of restrictive measures on the mental health, such as depression, anxiety and stress were already described [30, 31]. we can see an immediate improvement upon relaxation of measures. Increased loneliness can be attributed to social isolation, remote work or education, disruption of social activities. In other studies, similar increases of loneliness were also discovered [32]. Those findings highlight the importance of assessing the malign effects on population health not only from the infection perspective, but also from the mental health point of view, when developing containment policies.

One of the major strengths of this study is the relatively large sample size and representativeness of the cohort, which was preserved at each visit with implementation of weighting techniques, allowing us to observe and investigate the progression of the pandemic and pandemic-related matters on the scale of a whole country.

One of the challenges in longitudinal cohort studies is attrition during follow-up [33]. Continued study participation is of importance to maintain the statistical power of research and facilitate representativeness of study findings. The follow-up rate of 85% is considered good [34, 35]. In the CON-VINCE study, the attendance rate remained relatively high throughout the study, not falling below 83% (at visit 4). This drop may be explained by respondents losing incentive after participating in four repeated visits with only 2 weeks apart, along with entering into the summer months and changes in confinement measures. However, the attrition rate decreased for visit 5 (91% of respondents participated in that visit), which took place 40 weeks after the initial visit. The fact that there was no biosampling performed during that visit may also explain the higher number of active participants.

This observation highlights that several factors may have a noticeable effect on the attendance rate (frequency of repeated visits, procedures scheduled). Hence, study schemes where the visits are more spread out may bring benefits in terms of improving attendance rates [35]. Demographic analysis of drop-outs provides insight into identifying the groups of participants who may require additional encouragement in order to mitigate the possibility of them dropping out, when conducting other studies.

The final dropout rate at visit 6 was 15% (1,578 out of 1,865 participants attended last visit). This low rate of non-attendance may potentially indicate a high motivation of the study population.

There are some limitations to this study that should be acknowledged. Firstly, the period prevalence of COVID-19 in this study might have been underestimated due to waning antibodies [36]. Secondly, the comparisons with the official figures were made under the assumption that all cases reported by governmental services were adults, as we could not exclude children from the figures due to unavailability of such public data.

## Conclusion

To our knowledge, the CON-VINCE study is one of the few population-representative studies with deep phenotyping, biosampling, and longitudinal follow-up of the participants during COVID-19 pandemic. The data collected in CON-VINCE study allowed obtaining insights into the infection spread in Luxembourg, immunity build-up and the impact of the pandemic on psychological wellbeing of the population. Moreover, the study holds great translational potential, as samples stored at the biobank, together with self-reported questionnaire information, can be exploited in further research in defining vaccination strategies, analyses of the spread of SARS-CoV-2 variants and addressing challenges such as long COVID and consequences of self-isolation.

## Supplementary Information

**Additional file 1: Supplementary Table 1.** Detailed information provided by participants in the online survey

**Additional file 2:** **Supplementary Table 2.** Prevalence of COVID-19 symptoms at visit 6 (weighted)

**Additional file 3: Supplementary Table 3.** Comparison of dropouts and non-dropouts

## Data Availability

The dataset for this manuscript is not publicly available as it is linked to the CON-VINCE Study and its internal regulations. Any requests for accessing the dataset can be directed at *con-vince@lih.lu.* All data of the manuscript will be provided upon reasonable request and approval by the ethics committee.
