# Correction: Creation of a pandemic memory by tracing COVID-19 infections and immunity in Luxembourg (CON-VINCE)

**DOI:** 10.1186/s12879-025-11160-6

**Published:** 2025-07-03

**Authors:** Olena Tsurkalenko, Dmitry Bulaev, Marc Paul O’Sullivan, Chantal Snoeck, Soumyabrata Ghosh, Alexey Kolodkin, Basile Rommes, Piotr Gawron, Carlos Vega Moreno, Clarissa P. C. Gomes, Anne Kaysen, Jochen Ohnmacht, Valerie E. Schröder, Lukas Pavelka, Guilherme Ramos Meyers, Laure Pauly, Claire Pauly, Anne-Marie Hanff, Max Meyrath, Anja Leist, Estelle Sandt, Gloria A. Aguayo, Magali Perquin, Manon Gantenbein, Tamir Abdelrahman, Jochen Klucken, Venkata Satagopam, Christiane Hilger, Jonathan Turner, Michel Vaillant, Joëlle V. Fritz, Markus Ollert, Rejko Krüger, on behalf of the CON-VINCE consortium and the ORCHESTRA working group, Geeta Acharya, Pinar Alper, Wim Ammerlaan, François Ancien, Ariane Assele-Kama, Christelle Bahlawane, Katy Beaumont, Nadia Beaupain, Lucrèce Beckers, Camille Bellora, Fay Betsou, Luc Biver, Sandie Boly, Dirk Brenner, Henry-Michel Cauchie, Eleftheria Charalambous, Emilie Charpentier, Estelle Coibion, Sylvie Coito, Delphine Collart, Manuel Counson, Brian De Witt, Antonelle Di Pasquale, Olivia Domingues, Claire Dording, Jean-Luc Dourson, Bianca Dragomir, Tessy Fautsch, Jean-Yves Ferrand, Thibault Ferrandon, Ana Festas Lopes, Guillaume Fournier, Laura Georges, Stéphane Gidenne, Enrico Glaab, Borja Gomez Ramos, Vyron Gorgogietas, Jérôme Graas, Valentin Groues, Wei Gu, Gael Hamot, Maxime Hansen, Linda Hansen, Lisa Hefele, Laurent Heirendt, Ahmed Hemedan, Estelle Henry, Margaux Henry, Eve Herkenne, Sascha Herzinger, Laetitia Huiart, Alexander Hundt, Judith Hübschen, Gilles Iserentant, Philipp Jägi, Piyapong Khurmin, Fédéric Klein, Tommy Klein, Stéphanie Kler, Pauline Lambert, Jacek Jaroslaw Lebioda, Sabine Lehmann, Marie Leick, Morgane Lemaire, Andrew Lumley, Annika Lutz, João Manuel Loureiro, Monica Marchese, Tainà Marques, François Massart, Patrick May, Maura Minelli, Alessandra Mousel, Maeva Munsch, Sophie Mériaux, Friedrich Mühlschlegel, Mareike Neumann, Trang Nguyen, Beatrice Nicolai, Leslie Ogorzaly, Christiane Olesky, Christian Penny, Achilleas Pexaras, Palma di Pinto, Marie France Pirard, Jean-Marc Plesseria, Armin Rauschenberger, Lucie Remark, Antonio Rodriguez, Kirsten Rump, Bruno Santos, Aurélie Sausy, Margaux Schmitt, Christiane Schmitt, Reinhard Schneider, Serge Schumacher, Alexandra Schweicher, Sneeha Seal, Jean-Yves Servais, Florian Simon, Amna Skrozic, Kate Sokolowska, Lara Stute, Hermann Thien, Stéphane Toll, Noua Toukourou, Christophe Trefois, Johanna Trouet, Nguyen Trung, Daniela Valoura Esteves, Charlène Verschueren, Maharshi Vyas, Claus Vögele, Cécile Walczak, Xinhui Wang, Femke Wauters, Bernard Weber, Emilie Weibel, Tania Zamboni

**Affiliations:** 1 Luxembourg Institute of Health, Strassen, Luxembourg; 2 University of Luxembourg, Esch-Belval, Luxembourg; 3 Centre Hospitalier de Luxembourg, Luxembourg, Luxembourg; 4 Maastricht University Medical Centre, Maastricht, The Netherlands; 5 Laboratoire National de Santé, Dudelange, Luxembourg

**Correction: Tsurkalenko et al. BMC Infectious Diseases (2024) 24:17**

10.1186/s12879-024-09055-z

Following publication of the original article [1], the authors realised that they failed to add Armin Rauschenberger to the authorship list. Armin Rauschenberger contributed to the harmonization and initial stages of data analysis and his conceptual input on the analytical approach was crucial for this work. His analysis code contributed to the final analysis of data. Due to him changing his position and an additional change in the project management team he was not listed as a co-author.

The original article has been corrected and Armin Rauschenberger is added as the 9th author.

The Author Contribution section was also updated to list A.R.’s contributions to:

- Data management and data curation, writing data management section of the manuscript.
- Partial data analysis.

## References

[1] Tsurkalenko, et al. BMC Infect Dis. 2025;24:179. 10.1186/s12879-024-09055-z 10.1186/s12879-024-09055-z PMC10858600 38336649

