# Supplementary material for: Creation of a pandemic memory by tracing COVID-19 infections and immunity in Luxembourg (CON-VINCE)
Source: BMC Infect Dis. 2024 Feb 9;24:179. doi: 10.1186/s12879-024-09055-z (PMC10858600; doi:10.1186/s12879-024-09055-z)
Supplement: Supplementary file 1 — Additional file 1: Supplementary Table 1. Detailed information provided by participants in the online survey [file 12879_2024_9055_MOESM1_ESM.docx]

Supplementary Table 1. Detailed information provided by participants in the online survey.

| Category | Details |
| --- | --- |
| Demographics | Age, gender, country of origin, place of residence, marital status, number of children, household composition, and the age of household members |
| Comorbidities and current medications | Cardiac, hepatic, metabolic, pulmonary, neurological, haematological, oncological, gynaecological history, allergies, smoking status |
| Socio-economic status | Educational level, professional background current employment status, income, home-ownership |
| Environmental conditions for home care | Quarantine and self-isolation information |
| Behavioural and psychological changes during the COVID-19 pandemic | Physical activity, frequency of leaving the house, alcohol consumption, compliance with the recommendations and restrictions issued by the Luxembourgish Government during the COVID-19 pandemic |
| Epidemiological data and travel history | Travelling to an area with confirmed SARS-CoV-2 infections before they participated in the study |
| COVID-19-related information | If participants had already been tested for the SARS-CoV-2, indicating the date and the result of the test; contact tracing information; symptoms |
| Vaccination status | Reasons for willingness/unwillingness to vaccinate, date number of dosage, type of vaccine, adverse events |
