# Supplementary material for: Creation of a pandemic memory by tracing COVID-19 infections and immunity in Luxembourg (CON-VINCE)
Source: BMC Infect Dis. 2024 Feb 9;24:179. doi: 10.1186/s12879-024-09055-z (PMC10858600; doi:10.1186/s12879-024-09055-z)
Supplement: Supplementary file 2 — Additional file 2: Supplementary Table 2. Prevalence of COVID-19 symptoms at visit 6 (weighted) [file 12879_2024_9055_MOESM2_ESM.docx]

Supplementary Table 2. Prevalence of COVID-19 symptoms at visit 6 (weighted).

| Symptoms | Total  *n* | Prevalence  (weighted)  % | Prevalence non-infected (weighted)  % | Prevalence infected (weighted)  % |
| --- | --- | --- | --- | --- |
| Depression ^a^ | 1570 | 22.1% | 21.9% | 23.8% |
| Anxiety ^a^ | 1572 | 28.6% | 27.6% | 34.4% |
| Fever | 1537 | 1.8% | 1.8% | 1.5% |
| Cough | 1565 | 4.4% | 4.3% | 5.4% |
| Loss of smell | 1572 | 1.3% | 0% | 7.8% |
| Loss of taste | 1572 | 0.7% | 0% | 3.9% |
| Loss of appetite | 1572 | 0.3% | 0.1% | 0.9% |
| Runny nose | 1572 | 3.2% | 3.1% | 4% |
| Sore throat | 1572 | 0.8% | 0.8% | 1.1% |
| Ear pain | 1572 | 0.6% | 0.5% | 1% |
| Swollen lymphatic nodes | 1572 | 0.3% | 0.3% | 0.6% |
| Wheezing while breathing | 1568 | 1.9% | 1.7% | 3.2% |
| Chest pain | 1568 | 0.6% | 0.7% | 0% |
| Shortness of breath | 1568 | 3% | 2.3% | 5.9% |
| Muscle pain | 1559 | 6.5% | 6.4% | 7.8% |
| Joint pain | 1559 | 9.4% | 9.1% | 11.5% |
| Fatigue | 1562 | 7.2% | 5.6% | 15.6% |
| Headache | 1566 | 2% | 1.7% | 3.5% |
| Vertigo | 1566 | 1% | 0.7% | 2.4% |
| Cramps/ Seizures | 1566 | 0.1% | 0.1% | 0.5% |
| Altered state of consciousness/ confusion | 1566 | 1% | 0.9% | 2.2% |
| Abdominal pain | 1567 | 1% | 0.7% | 2.6% |
| Nausea/ Vomiting | 1567 | 0.6% | 0.4% | 1.4% |
| Diarrhoea | 1567 | 1.1% | 1.1% | 0.8% |
| Skin rash | 1564 | 2.4% | 1.8% | 4.8% |
| Sleep difficulties | 1564 | 16.2% | 15.4% | 19.6% |
| Hair loss | 1564 | 2.9% | 2.5% | 4.9% |
| Palpitation | 1555 | 2.4% | 1.6% | 6.3% |
| Memory loss | 1569 | 0% | 0% | 0% |
| Presence of COVID-19 symptoms ^b^ | 1578 | 49.2% | 47% | 59.2% |

^a^ Derived variables. Depression was assumed when CES-D scale total score >=16. Anxiety was assumed, when GAD-7 scale total score >=5
^b^ If the status of at least one of 29 symptoms listed was known at visit 6, participants were included in this summar*y*
