# Supplementary material for: Creation of a pandemic memory by tracing COVID-19 infections and immunity in Luxembourg (CON-VINCE)
Source: BMC Infect Dis. 2024 Feb 9;24:179. doi: 10.1186/s12879-024-09055-z (PMC10858600; doi:10.1186/s12879-024-09055-z)
Supplement: Supplementary file 3 — Additional file 3: Supplementary Table 3. Comparison of dropouts and non-dropouts [file 12879_2024_9055_MOESM3_ESM.docx]

Supplementary Table 3. Comparison of dropouts and non-dropouts.

|  | Non-dropouts | Dropouts | Unadjusted p-value | Adjusted p-value |
| --- | --- | --- | --- | --- |
|  | Mean (SD) | Mean (SD) |  |  |
| Age, years | 49 (15) | 43 (15) | <0.01** | <0.01** |
| Gender | Freq. (Proportion) | Freq. (Proportion) | 0.027* | 0.08 |
| Male | 793 (50%) | 124 (43%) |  |  |
| Female | 783 (50%) | 163 (57%) |  |  |
| Education level | Freq. (Proportion) | Freq. (Proportion) | 0.041* | 0.124 |
| University degree | 650 (41%) | 115 (40%) |  |  |
| Secondary Education - Technical system | 376 (24%) | 87 (30%) |  |  |
| Secondary Education - Classical system | 247 (16%) | 48 (17%) |  |  |
| Other type of degree | 223 (14%) | 23 (8%) |  |  |
| No formal degree | 47 (3%) | 7 (2%) |  |  |
| Fundamental Education | 35 (2%) | 7 (2%) |  |  |

* Statistical significance at α=0.05 confidence level
 ** Statistical significance at α=0.01 confidence level
